# Pan-cancer predictions of transcription factors mediating aberrant DNA methylation

**DOI:** 10.1186/s13072-022-00443-w

**Published:** 2022-03-24

**Authors:** Dylane Detilleux, Yannick G. Spill, Delphine Balaramane, Michaël Weber, Anaïs Flore Bardet

**Affiliations:** UMR7242 Biotechnology and Cell Signaling, CNRS, University of Strasbourg, 67412 Illkirch, France

**Keywords:** Bioinformatics, DNA methylation, Cancer, Transcription factors

## Abstract

**Background:**

Aberrant DNA methylation is a hallmark of cancer cells. However, the mechanisms underlying changes in DNA methylation remain elusive. Transcription factors initially thought to be repressed from binding by DNA methylation, have recently emerged as being able to shape DNA methylation patterns.

**Results:**

Here, we integrated the massive amount of data available from The Cancer Genome Atlas to predict transcription factors driving aberrant DNA methylation in 13 cancer types. We identified differentially methylated regions between cancer and matching healthy samples, searched for transcription factor motifs enriched in those regions and selected transcription factors with corresponding changes in gene expression. We predict transcription factors known to be involved in cancer as well as novel candidates to drive hypo-methylated regions such as FOXA1 and GATA3 in breast cancer, FOXA1 and TWIST1 in prostate cancer and NFE2L2 in lung cancer. We also predict transcription factors that lead to hyper-methylated regions upon transcription factor loss such as EGR1 in several cancer types. Finally, we validate that FOXA1 and GATA3 mediate hypo-methylated regions in breast cancer cells.

**Conclusion:**

Our work highlights the importance of some transcription factors as upstream regulators shaping DNA methylation patterns in cancer.

**Supplementary Information:**

The online version contains supplementary material available at 10.1186/s13072-022-00443-w.

## Background

DNA methylation is the most studied epigenetic modification in cancer [1]. Hyper-methylation of CpG island promoters of tumor suppressor genes associated to gene silencing is a hallmark of cancer [2]. More recently, regions of global hypo-methylation were identified in cancer as long partially methylated domains observed at late-replicating lamina-associated domains [3, 4].

Methylation of DNA occurs on cytosines mostly within CpG dinucleotides and is catalyzed by DNA methyltransferases: DNMT1, DNMT3a and DNMT3b. DNA methylation is abundant throughout the genome except at CpG islands that are constitutively protected from DNA methylation. Initially, DNA methylation has been described as a transcriptional repressor, where the presence of DNA methylation at gene promoters would block transcription factor (TF) binding leading to gene silencing [5, 6]. More recently, genome-wide studies showed that active regulatory elements bound by TFs invariably correlate with focal regions of low methylation and in contrast to the classical model, showed that TF binding could induce active demethylation mediated by the TET enzymes [7–10]. Several studies have investigated the interplay between TF binding and DNA methylation that we recently reviewed [11]. This strong anti-correlation between patterns of TF binding and DNA methylation therefore enables to infer active regulatory regions using DNA methylation data. However, it does not provide causal information about which of TF binding or DNA methylation regulates one another.

Despite extensive studies, the mechanisms leading to the accumulation of aberrant DNA methylation patterns in cancer are still poorly understood. Previous studies have correlated DNA methylation and gene expression changes to identify enhancers and their target genes [12–17]. Yao et al. studied ten cancer types and developed a method that correlated changes in DNA methylation of distal cytosines with changes in gene expression and then used 145 TF motifs to infer TF regulators [13]. Rhie et al. followed by Mullen et al. studied four cancer types and developed a method that correlated changes in DNA methylation of distal regulatory elements with changes in gene expression and focused on TFs [15, 17]. Fleischer et al. studied breast cancer and correlated DNA methylation changes of CpGs with gene expression and integrated TF-binding sites to identify transcriptional networks regulated by DNA methylation [16]. However, none of those studies validated experimentally that the predicted TFs drive changes in DNA methylation. Furthermore, recent profiling of chromatin-accessible regions marking TF occupancy in primary cancer samples correlated them to hypo-methylated regions suggested to be driven by key TFs [18]. However, investigating TF binding in primary cancer samples at a large scale remains challenging due to technical limitations.

Here, we exploited the massive amount of primary DNA methylation datasets from The Cancer Genome Atlas (TCGA) to predict TFs driving aberrant DNA methylation in cancer. We first identified differentially methylated regions (DMRs) between cancer and healthy samples. We performed a TF motif enrichment analysis to predict TF binding in DMRs. We then integrated matching TF expression data to distinguish TFs expected to drive DNA methylation changes in cancer. Finally, we validated our predictions in breast cancer cells and showed that FOXA1 and GATA3 indeed mediate DNA hypo-methylation.

## Results

### Processing of the TCGA methylation data

To study DNA methylation changes in cancer, we retrieved 8425 raw methylation datasets, generated from Illumina Infinium HumanMethylation450 BeadChip (HM450), for 32 available cancer types from the TCGA resource. We processed the data with the ChAMP pipeline [19, 20] and performed normalization using noob [21] as implemented in minfi [22, 23].

**Fig. 1:**
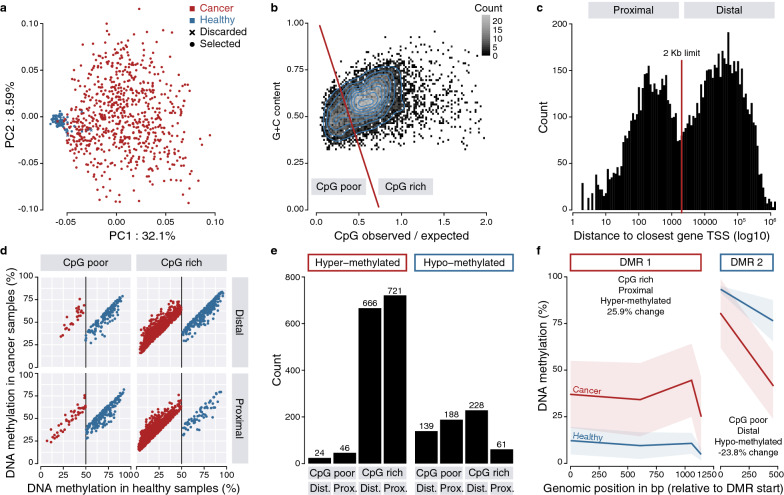
Identification of BRCA DMRs. **a** Principal component analysis of the BRCA cancer (red) and healthy (blue) samples and visualization of the samples discarded using a quadratic discriminant analysis (cross). **b** CpG and G + C content of BRCA DMRs. Density lines are represented in blue. Two categories, CpG-poor and CpG-rich, were defined according to a threshold following *y* = − 1.4(x − 0.38) + 0.51. **c** Distance of BRCA DMRs to their closest gene TSS. Two categories, proximal and distal, were defined according to a 2 kilobase (Kb) threshold. **d** Levels of methylation in hyper-methylated BRCA DMRs (red) or hypo-methylated DMRs (blue) in cancer versus healthy samples in the different categories. Only DMRs with at least 20% methylation change and a starting methylation mean in healthy samples above 50% for DMRs hypo-methylated in cancer and below 50% for hyper-methylated ones are shown. **e** Number of BRCA hyper- or hypo-methylated DMRs in the different categories. **f** Example of hyper- and hypo-methylated BRCA DMRs

We trained a quadratic discriminant analysis aiming to classify the cancer and healthy samples in two groups for each cancer type and discarded samples that were misclassified (Fig. 1a). Based on the number and dispersion of samples, we retained 13 cancer types for further analysis: bladder urothelial carcinoma (BLCA), breast invasive carcinoma (BRCA), cholangiocarcinoma (CHOL), colon adenocarcinoma (COAD), head and neck squamous cell carcinoma (HNSC), kidney renal clear cell carcinoma (KIRC), kidney renal papillary cell carcinoma (KIRP), liver hepatocellular carcinoma (LIHC), lung adenocarcinoma (LUAD), lung squamous cell carcinoma (LUSC), prostate adenocarcinoma (PRAD), thyroid carcinoma (THCA) and uterine corpus endometrial carcinoma (UCEC) (Additional file 1: Table S1). In the case of BRCA, representative of other cancer types, we observe distinct clustering of the healthy and cancer samples and an expected broader heterogeneity of cancer samples (Fig. 1a). The results of this analysis can be visualized interactively for all cancer types on our webserver http://bardet.u-strasbg.fr/cancermethtf/ in the section “Data”.

### Identification of differentially methylated regions in cancer

We identified DMRs between the cancer and healthy samples for each cancer type by first calling differentially methylated cytosines using limma [24] and then DMRs using a modified version of DMRcate [25]. Briefly, we only used cytosines not located in exons, which are not expected to contain TF-binding sites, and searched for DMRs containing at least two significant CpGs that were consistently hypo- or hyper-methylated.

We further classified DMRs according to their genomic features. Observation of the DMRs’ CpG and G + C contents revealed two distinct categories that we termed CpG-poor and CpG-rich (Fig. 1b). Observation of the distance of the DMRs to their closest gene transcription start site (TSS) also revealed two distinct categories that we termed proximal and distal (Fig. 1c). These features enable us to minimize the possible biases of the subsequent TF motif analysis due to the preference of some TF to bind specific genomic locations.

We then selected DMRs with a minimum length of 200 bp, expected for TF-binding sites, at least 20% methylation change and a starting methylation mean in healthy samples above 50% for DMRs hypo-methylated in cancer and below 50% for hyper-methylated ones (Fig. 1d). Finally, we took advantage of recently available chromatin accessibility ATAC-seq data in TCGA cancer samples [18] expecting putative TF-binding sites to be located in open chromatin ATAC-seq peaks. Therefore, we further selected hypo-methylated DMRs in cancer if they overlapped at least one ATAC-seq peak in the corresponding cancer samples and if hyper-methylated DMRs did not overlap any peak.

The vast majority of DMRs were hyper-methylated and located in CpG-rich regions (Fig. 1e), which was expected since the TCGA methylation array probes are enriched at gene promoters [26], usually CpG-rich, and changes in DNA methylation in cancer have previously been described as hyper-methylated in gene promoters. A substantial number of DMRs were also found as hypo-methylated including in CpG-poor regions (Fig. 1e), which likely represent enhancer regulatory regions bound by TFs. Examples of a hyper-methylated CpG-rich proximal DMR, i.e., promoter and a hypo-methylated CpG-poor distal DMR are shown (Fig. 1f). The results of these analyses can be visualized interactively for all cancer types on our webserver http://bardet.u-strasbg.fr/cancermethtf/ in the section “Differentially methylated regions”.

### Prediction of transcription factors driving DMRs across cancer types

In order to identify TFs driving DNA methylation changes in cancer, we search for potential TF-binding sites in DMRs. TFs bind to DNA through the recognition of short DNA sequences called motifs. We therefore performed an enrichment analysis of known TF motifs using our recently developed approach TFmotifView [27]. We extracted 4928 TF motifs from a manually annotated review [28], that we could group by similarity into 434 clusters and that represent 1048 distinct TFs. TF motif logos and clusters can be visualized on our webserver http://bardet.u-strasbg.fr/cancermethtf/ in the section “TF motif clusters”. Since hundreds of thousands of TF motif occurrences can be found over the genome, we computed an enrichment of how many of our DMRs contain at least one occurrence of each motif compared to control regions with similar genomic features or contrasted hypo-methylated DMRs with hyper-methylated DMRs from the same category. We then derived a hypergeometric *p*-value for each motif enrichment.

**Fig. 2:**
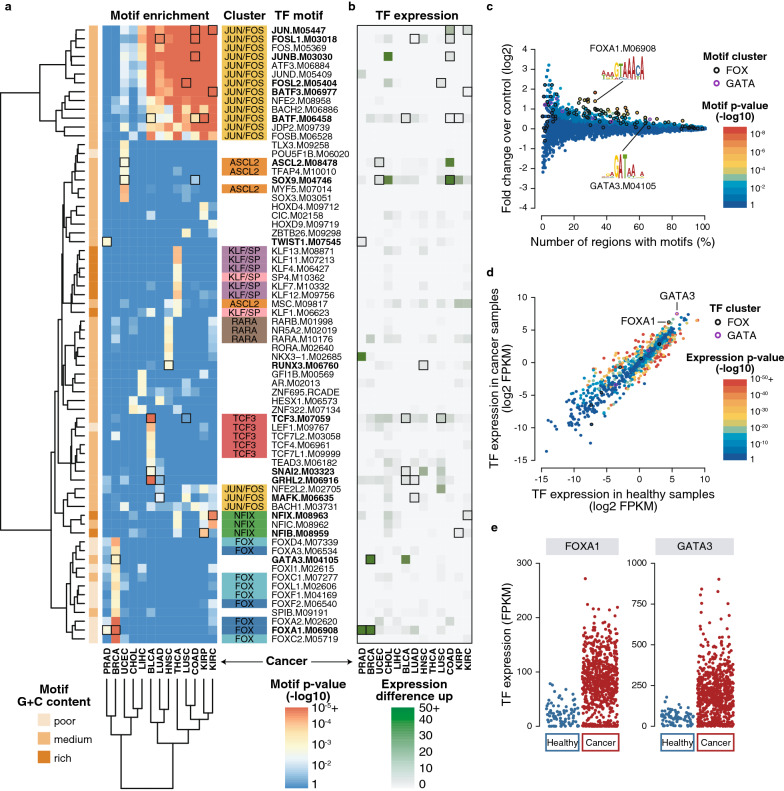
TF motif enrichment in hypo-methylated, CpG-poor, distal DMRs. **a** Pan-cancer motif enrichment. Heatmap of best enriched motifs across all cancer types using a *p*-value threshold of 10^–3^ and selecting one motif per TF using the best *p*-value summed across all cancers. Motif cluster and CpG content are shown. **b** Pan-cancer TF expression. Heatmap of the expression of corresponding TFs using positive mean FPKM difference between cancer and healthy samples. Motifs highlighted in bold with black squares have matching motif enrichment and TF upregulation. **c** BRCA motif enrichment. Motif enrichment in BRCA DMRs corresponding to the BRCA column in **a**. Motif *p*-values (point color) are computed using an hypergeometric test using the number of regions that have at least a motif compared to the fold enrichment over control regions. Each point represents one of the 4928 motifs used. FOX and GATA clusters are highlighted (including several FOXA1 or GATA3 motif points). **d** BRCA TF expression. TF expression enrichment in cancer compared to healthy samples (log2 mean FPKM). Each point represents one of the 1048 TFs used colored according to their differential expression *p*-value. FOX and GATA clusters are highlighted. **e** Expression of FOXA1 and GATA3 TFs. Dot plot showing all samples FPKM values for FOXA1 and GATA3 in cancer compared to healthy samples corresponding to the mean value shown in **d**

We searched for TF motifs in DMRs from all genomic categories and first focused on the hypo-methylated DMRs located in CpG-poor regions distal from genes TSS representing putative enhancer regions compared to control regions. Across almost all cancer types, the motifs from the cluster JUN/FOS were highly enriched except for BRCA and PRAD (Fig. 2a). Those TFs compose the Activator Protein 1 (AP-1) family that is involved in differentiation, proliferation, apoptosis and well known in tumorigenesis [29].

Several other TF motifs, sometimes from the same motif cluster and/or TF family, were enriched in specific cancer types, for example the FOX cluster in BRCA (Fig. 2a). In order to disentangle which specific TF among its motif cluster could drive the cancer DMRs, we integrated matching expression available from TCGA. We hypothesized that hypo-methylated DMRs, methylated in healthy samples and unmethylated in cancer samples, could be regulated in trans by TFs not or lowly expressed in healthy samples and overexpressed in cancer samples therefore binding specifically and driving hypo-methylation in cancer. We then searched for TFs whose expression was upregulated in the cancer samples compared to the healthy samples (Fig. 2b) and selected TFs that have both their motif enriched and higher expression in cancer (Fig. 2a,b**,** black squares). The two most enriched TF motifs with matching upregulation were FOXA1 and GATA3 in BRCA (motif *p*-value < 10^–5^ and < 10^–3^, respectively; expression *p*-value < 10^–20^ and < 10^–27^, respectively). The FOX and GATA motif clusters, containing many putative motifs, represented most of the motifs enriched in BRCA hypo-methylated DMRs located in CpG-poor regions distal from genes TSS (Fig. 2c). Out of their corresponding TFs, *FOXA1* and *GATA3* genes were the most upregulated in BRCA cancer samples (Fig. 2d,e). Both FOXA1 and GATA3 TFs are known markers in breast cancer [30].

When searching for motifs enriched in hypo-methylated CpG-poor DMRs either distal or proximal, the following TFs showed both motif enrichment and TF upregulation (Fig. 2a,b and Additional file 1: Figure S1): FOXA1, GATA3, RFX5 and TFAP2A in BRCA; TCF3, GRHL2, SNAI2, PATZ1 and BATF in BLCA; JUN, JUNB, FOSL1 and BATF in COAD; RUNX3, FOSL1 and BATF in HNSC; NFIX, BACH1, JUN, BATF and BATF3 in KIRC; NFIB and BATF in KIRP; FOXA1 and FOXA2 in LIHC; HSF1, FOSL1 and BATF in LUAD; NFE2L2, HSF1, GRHL1, GRHL2, TFAP2A, TFAP2C and FOSL2 in LUSC; FOXA1 and TWIST1 in PRAD; ASCL2, SOX9, CEBPB and ESRRA in UCEC. Only very few hyper-methylated DMRs were identified as CpG-poor both distal and proximal and therefore only few TFs showed both motif enrichment and TF downregulation (Additional file 1: Figure S1): KLF5 in BRCA; FOXA2 in CHOL; ARID5B in KIRC; STAT3 in PRAD; RXRG, EGR2 and ZNF263 in UCEC. Many of those TFs have previously been involved in cancer.

**Fig. 3:**
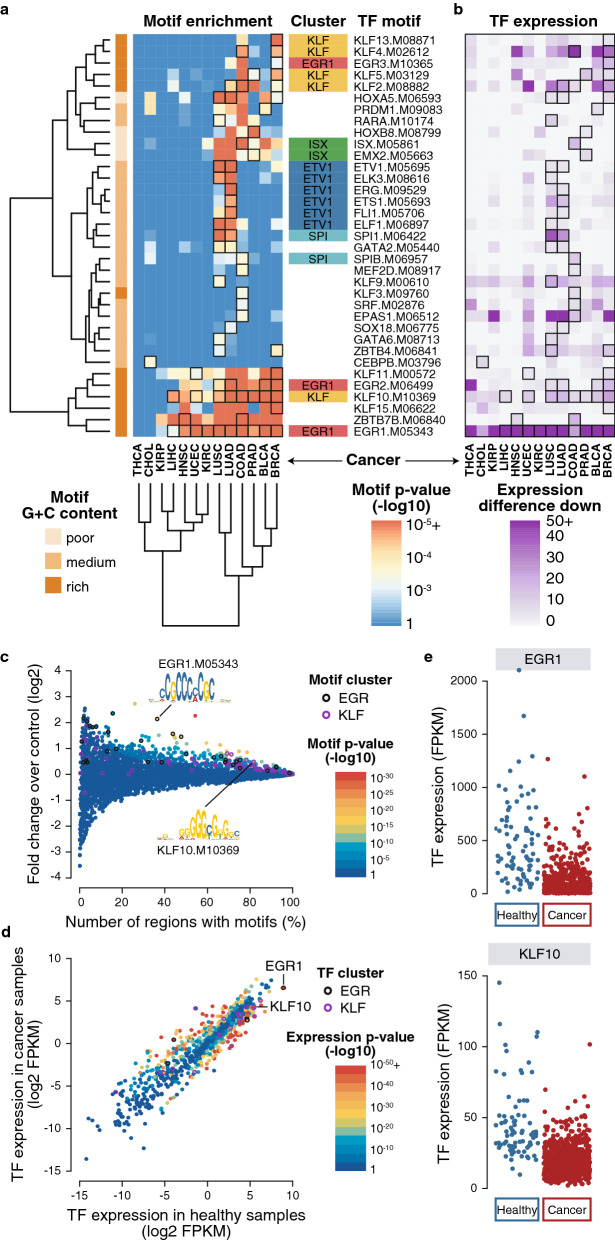
TF motif enrichment in hyper-methylated, CpG-rich, distal DMRs. **a** Pan-cancer motif enrichment. Heatmap of best enriched motifs across all cancer types using a *p*-value threshold of 10^–3^ and selecting one motif per TF using the best *p*-value summed across all cancers. Only motifs with matching expression downregulation are shown. Motif cluster and CpG content are shown. **b** Pan-cancer TF expression. Heatmap of the expression of corresponding TFs using negative mean FPKM difference between cancer and healthy samples. All motifs represented here have matching expression and TF downregulation. **c** BRCA motif enrichment. Motif enrichment in BRCA DMRs corresponding to the BRCA column in **a.** Motif *p*-values (point color) are computed using an hypergeometric test using the number of regions that have at least a motif compared to the fold enrichment over control regions. Each point represents one of the 4928 motifs used. EGR and KLF clusters are highlighted (including several EGR1 or KLF10 motif points). **d** BRCA TF expression. TF expression enrichment in cancer compared to healthy samples (log2 mean FPKM). Each point represents one of the 1048 TF used colored according to their differential expression *p*-value. EGR and KLF clusters are highlighted. **e** Expression of EGR1 and KLF10 TFs. Dot plot showing all samples FPKM values for EGR1 and KLF10 in cancer compared to healthy samples corresponding to the mean value shown in **d**

When searching for motifs enriched in CpG-rich DMRs, likely to contain different TF motifs due to their distinct sequence content, we contrasted the motif content of hypo- versus hyper-methylated DMRs. Hypo-methylated DMRs were enriched for similar motifs in CpG-rich categories than in CpG-poor categories (Additional file 1: Figure S2). Hyper-methylated CpG-rich DMRs were consistently enriched in G + C-rich low complexity motifs such as EGR1 or KLF in most cancer types (Fig. 3a,b and Additional file 1: Figure S2). Both EGR and KLF motif clusters, containing many putative motifs, were enriched in BRCA hyper-methylated DMRs (Fig. 3c). Out of their corresponding TFs, *EGR1* and *KLF10* genes were downregulated in BRCA cancer samples (Fig. 3d,e). EGR1 has been shown to have significant tumor suppressor properties in many types of cancer [31, 32] and different KLF TFs have been involved in a large number of cancers [33].

The results of all motif analyses for DMRs in all categories can be visualized interactively for all cancer types on our webserver http://bardet.u-strasbg.fr/cancermethtf/ in the section “TF motif enrichment and expression”.

### Correlation between DNA methylation and binding of FOXA1 and GATA3 in breast cancer cell lines

**Fig. 4:**
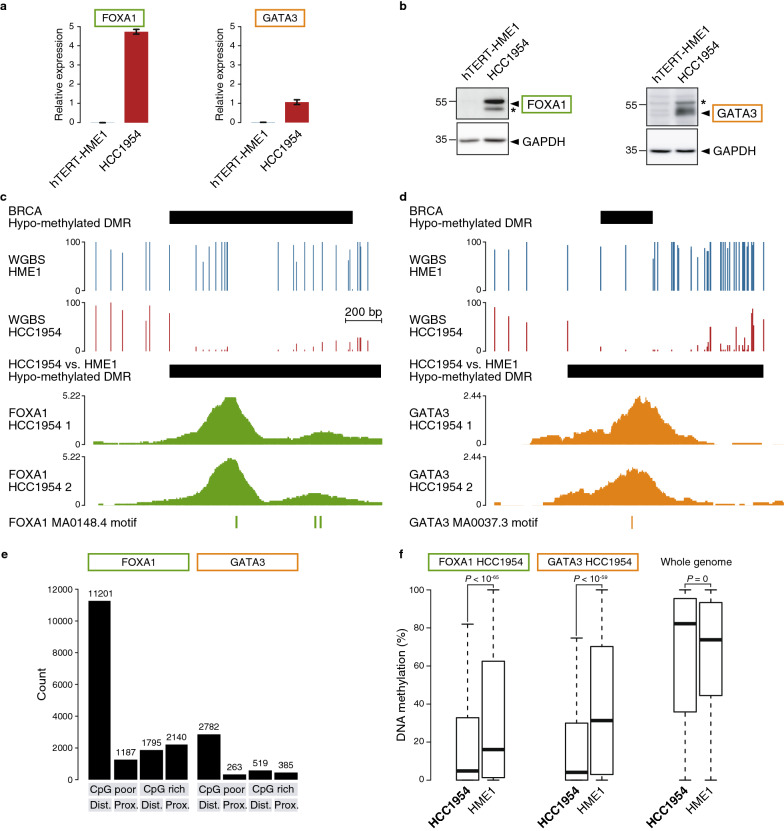
FOXA1 and GATA3 bind hypo-methylated regions in HCC1954 breast cancer cells. **a** Expression levels of FOXA1 and GATA3 in hTERT-HME1 and HCC1954 cells by RT-qPCR (mean ± SEM, *n* = 3 independent replicates; relative to RPL13A expression). **b** Protein levels of FOXA1 and GATA3 in hTERT-HME1 and HCC1954 cells by western blotting. GAPDH was used as an internal control for equal loading. Stars indicate nonspecific bands. **c** Genome browser view (chr14:75521286-75521809) of an hypo-methylated DMR in HCC1954 breast cancer cells compared to hTERT-HME1 normal cells matching a TCGA BRCA hypo-methylated DMR, FOXA1 ChIP-seq signal in HCC1954 cells in two replicates and location of FOXA1 motifs. **d** Genome browser view (chr2:27209983-27210462) as in **c** matching a GATA3 binding sites and motif. **e** Number of FOXA1 and GATA3 binding peaks in the different genomic categories: CpG-poor distal from gene TSS, CpG-poor proximal, CpG-rich distal or CpG-rich proximal. **f** Boxplots of mean DNA methylation in HCC1954 and hTERT-HME1 cells in 200 bp windows around FOXA1 or GATA3 HCC1954 peak summits that contain at least 2 CpGs and overlapping a matching FOXA1 or GATA3 motif (FOXA1 *n* = 4709; GATA3 *n* = 1671) or in all 200 bp consecutive windows along the hg38 genome containing at least 2 CpGs (*n* = 5,867,466). Wilcoxon *p*-values are indicated

We next set out to validate experimentally if the TFs FOXA1 and GATA3 drive changes in DNA methylation in common breast cancer cell lines. We used the HCC1954 cell line derived from a primary breast tumor and the hTERT-HME1 cell line as normal mammary epithelial cells. These cell lines have been derived from women of similar age (61 and 53 years old, respectively) and hTERT-HME1 cells were derived from a healthy donor and have a normal karyotype. HCC1954 cells show high expression of *FOXA1* and *GATA3* at both mRNA and protein levels compared to hTERT-HME1 normal cells (Fig. 4a,b) and therefore recapitulate well their expression observed in the TCGA BRCA samples. We performed whole genome bisulfite sequencing (WGBS) on those cell lines (Additional file 1: Figure S3a and Table S2) and searched for DMRs using DSS [34] (Additional file 1: Table S3). We identified 145,826 hypo-methylated DMRs and 121,090 hyper-methylated DMRs in HCC1954 cancer cells compared to hTERT-HME1 normal cells. Of the TCGA BRCA DMRs and although the data have more limited genome coverage, 144 out of 616 hypo-methylated DMRs (23%) and 1013 out of 1457 hyper-methylated DMRs (69%) overlapped hypo- or hyper-methylated DMRs in HCC1954 versus hTERT-HME1, respectively (see examples in Fig. 4c,d).

We then looked for FOXA1 and GATA3 binding sites in HCC1954 breast cancer cells by performing chromatin immunoprecipitation sequencing (ChIP-seq) using FOXA1 and GATA3 antibodies in two biological replicates each (Additional file 1: Table S4). After peak calling using peakzilla [35] and filtering out false-positive peaks due to genomic amplification in the HCC1954 breast cancer cells, we obtained 13,753 and 14,257 peaks for FOXA1 replicates samples and 2095 and 3751 peaks for GATA3 replicates samples (see examples in Fig. 4c,d and Additional file 1: Table S4). Since the ChIP signal correlated well between replicates (Additional file 1: Figure S4a), we merged peak regions for further analyses yielding 16,323 peak regions for FOXA1 and 3,949 peak regions for GATA3. Although some binding sites were shared between FOXA1 and GATA3, the majority were distinct (Additional file 1: Figure S4b). Further, 53% of FOXA1 peak regions and 69% of GATA3 peak regions contained the FOXA1 and GATA3 motifs, respectively, which is a usual fraction found in TF ChIP-seq peaks. We next categorized the peak regions according to their genomic features and found that the majority of FOXA1 and GATA3 binding sites were located in CpG-poor regions distal from gene TSS, as expected for TFs binding distal regulatory regions (Fig. 4e and Additional file 1: Figure S4c,d).

Finally, we investigated the methylation patterns at FOXA1 and GATA3 binding sites. We could show that they were located in regions with low DNA methylation levels in HCC1954 cancer cells (Fig. 4f), expected for TF-binding sites in active regulatory regions. Furthermore, FOXA1 and GATA3 binding sites were significantly more methylated in hTERT-HME1 normal cells lacking FOXA1 and GATA3 expression (Fig. 4f and Additional file 1: Figure S4e). Indeed, we found that 20% and 23% of direct FOXA1 and GATA3 peak regions containing CpGs overlapped DMRs hypo-methylated in HCC1954 cancer cells compared to hTERT-HME1 normal cells (see examples in Fig. 4c, d). Of interest, 269 and 293 TCGA BRCA hypo-methylated DMRs have FOXA1 or GATA3 motifs, respectively, (representing 39% and 42% of all 695). Although we do not expect all motif occurrences in a genome to be bound, we found that 66 and 35 of direct FOXA1 and GATA3 peak regions in HCC1954 cells, respectively, overlapped those TCGA BRCA hypo-methylated DMRs (representing 24% and 12%; see examples in Fig. 4c,d).

Altogether, these data identify FOXA1 and GATA3 binding sites that correlate with DNA hypo-methylation in HCC1954 cancer cells. These regions represent putative regions where DNA hypo-methylation could be mediated by FOXA1 or GATA3 binding, although other FOX and GATA TFs are expressed in HCC1954 cells [4].

### FOXA1 and GATA3 mediate DNA hypo-methylation in HCC1954 cells

**Fig. 5:**
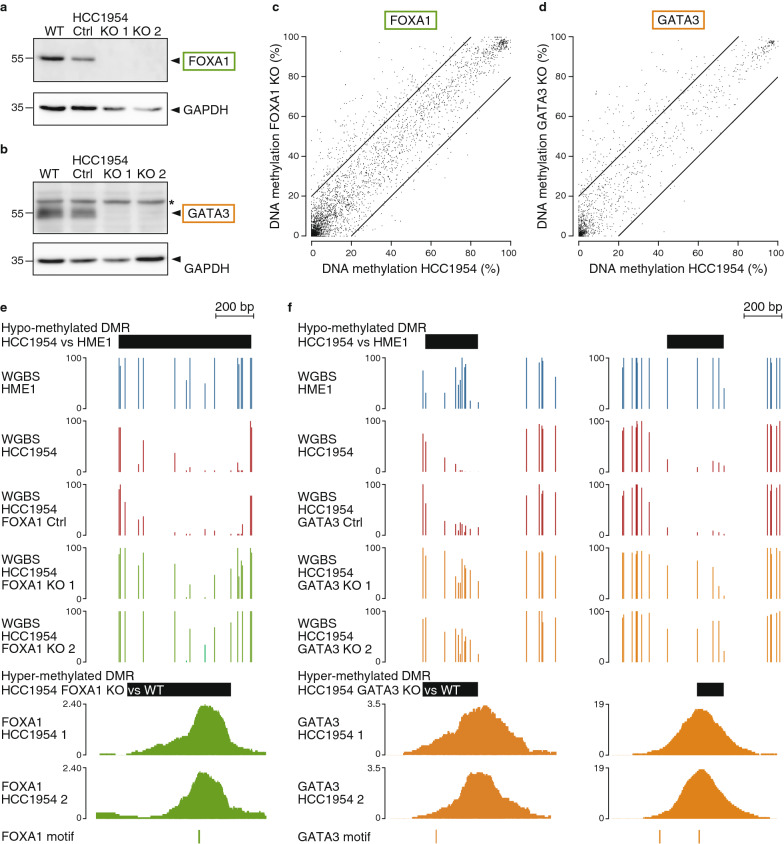
Gain of DNA methylation upon FOXA1 or GATA3 removal in HCC1954 breast cancer cells. **a** Western blot analysis of FOXA1 protein levels in parental HCC1954 cells (WT), a control clone (Ctrl) and two FOXA1 KO clones (KO1 and KO2). GAPDH was used as an internal control for equal loading. **b** Western blot analysis of GATA3 protein levels in parental HCC1954 cells (WT), a control clone (Ctrl) and two GATA3 KO clones (KO1 and KO2). GAPDH was used as an internal control for equal loading. Star indicates nonspecific bands. **c** DNA methylation levels in HCC1954 FOXA1 KO cells compared to HCC1954 cells (mean across samples) in 200 bp windows around FOXA1 peak summits that contain at least 2 CpGs and overlapping a matching FOXA1 motif (*n* = 4473). **d** DNA methylation levels in HCC1954 GATA3 KO cells compared to HCC1954 cells around GATA3 peak summits as in **c**. (*n* = 1598). **e** Genome browser view (chr19:16093263-16093982) of an hyper-methylated DMR in HCC1954 FOXA1 KO cells compared to HCC1954 cells matching hypo-methylated DMR in HCC1954 vs HME1 and FOXA1 binding sites and motif. **f** Genome browser views (chr1:214377303-214378552 and chr8:42296869-42299784) as in **e** of two hyper-methylated DMRs in HCC1954 GATA3 KO cells compared to HCC1954 cells

We next sought to determine if FOXA1 and GATA3 are causally involved in driving hypo-methylation in HCC1954 breast cancer cells. To test this, we used CRISPR/Cas9 to knockout (KO) FOXA1 or GATA3 in HCC1954 cancer cells. We generated two independent KO lines which were validated by Sanger sequencing and Western blot (Fig. 5a,b and Additional file 1: Figure S5a,b). We performed WGBS in two independent FOXA1 and GATA3 KO clones (Additional file 1: Fig. S3b, c and Table S2, 3) and could observe a gain of DNA methylation in FOXA1 or GATA3 KO cells compared to wildtype (WT) HCC1954 cells at FOXA1 or GATA3 binding sites, respectively (Fig. 5c–f and Additional file 1: Figure S5c-f). We found 82 FOXA1 binding peak regions located in FOXA1 KO hyper-methylated DMRs (see example in Fig. 5e) and 30 GATA3 binding peak regions located in GATA3 KO hyper-methylated DMRs (see examples in Fig. 5f), which we expect to result from a direct consequence of FOXA1 or GATA3 removal. We indeed find that FOXA1 or GATA3 binding sites are significantly enriched in FOXA1 or GATA3 KO hyper- over hypo-methylated DMRs (6.8-fold enrichment with hypergeometric *p*-value < 10^–13^ for FOXA1 and 15-fold with *p*-value < 10^–6^ for GATA3). Further, 49% of FOXA1 and 43% for GATA3 binding peak regions in KO hyper-methylated DMRs also overlapped HCC1954 hypo-methylated DMRs compared to hTERT-HME1 cells, which is significant over shuffled HCC1954 DMRs (4.4-fold enrichment with hypergeometric *p*-value < 10^–7^ for FOXA1 and 6.5-fold with *p*-value < 10^–3^ for GATA3). This shows that FOXA1 and GATA3 do maintain hypo-methylated regions in cancer compared to normal cells at a subset of their binding sites.

**Fig. 6:**
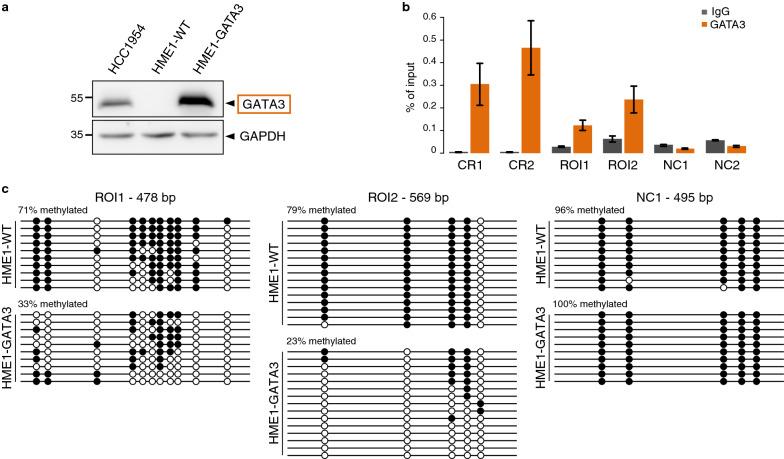
Loss of DNA methylation upon GATA3 overexpression in hTERT-HME1 normal cells. **a** Western blot analysis of GATA3 protein levels in HCC1954 cells and hTERT-HME1 cells stably transfected with a plasmid containing the GATA3 ORF (HME1-GATA3) or non-transfected (HME1-WT). GAPDH was used as a control for equal loading. **b** ChIP-qPCR analysis for GATA3 binding at two positive control regions CR1 and CR2 (regions unmethylated in hTERT-HME1 and HCC1954 cells with GATA3 motif and binding in HCC1954 cells), at two regions of interest ROI1 and ROI2 from Fig. 5f (regions methylated in hTERT-HME1 cells, unmethylated in HCC1954 cells, with GATA3 motif and binding in HCC1954 cells, and gaining methylation in GATA3 KO clones), and at two negative control regions NC1 (region methylated in hTERT-HME1 cells, unmethylated in HCC1954 cells with no GATA3 motif and binding in HCC1954 cells), NC2 (intergenic region unmethylated in hTERT-HME1 and HCC1954 cells with no GATA3 motif and binding in HCC1954 cells). ChIP was performed with antibody against GATA3 or a control IgG antibody. ChIP signals are represented as percentage input. Data represent mean ± SEM (*n* = 3 biological replicates). **c** Patterns of CpG methylation of ROI1, ROI2 and NC1 by bisulfite sequencing analysis in HME1 WT and HME1 overexpressing GATA3 (HME1-GATA3). White circles represent unmethylated CpGs and black circles represent methylated CpGs

Finally, we sought to determine if GATA3 is able to bind methylated DNA and induce demethylation in hTERT-HME1 normal cells that do not express *GATA3*. To this end, we generated hTERT-HME1 cells with stable overexpression of *GATA3* and validated it by Western blot (Fig. 6a). We then profiled GATA3 binding by ChIP-qPCR and observed binding at two regions of interest that are bound by GATA3 in HCC1954 cells and gain methylation in GATA3-KO HCC1954 cells (Figs. 5f, 6b). We then profiled DNA methylation at those sites by bisulfite sequencing and observed demethylation upon GATA3 overexpression in hTERT-HME1 cells (Fig. 6c). This shows that overexpressed GATA3 can indeed mediate DNA hypo-methylation in normal cells at sites bound in breast cancer cells.

## Discussion

In this study, we took advantage of the massive amount of primary DNA methylation datasets from TCGA and developed a computational approach to predict TFs driving aberrant DNA methylation in cancer. Due to the limited number of CpG probes present in the HM450 array covering 1.7% of the human genome and their bias toward gene promoters [26], we identified a majority of hyper-methylated DMRs located in CpG-rich regions. However, thanks to the CpG probes designed in putative enhancer regions, we could identify a substantial number of hypo-methylated DMRs.

To predict which TFs could drive those DMRs, we performed a comprehensive TF motif analysis. Since several TFs recognize similar motifs, we used TF gene expression to predict which specific TF could act in trans to mediate DMRs: we searched for downregulated TFs whose motifs were identified in hyper-methylated DMRs and for upregulated TFs in hypo-methylated DMRs. Based on this strategy, we did not consider some highly enriched TFs that did not display matching expression such as ZBTB14 in CpG-rich hyper-methylated DMRs in all cancers (except CHOL and THCA), confirming findings from a previous pan-cancer analysis [36]. However, we cannot exclude that TFs expressed at the same level in cancer and normal samples could also impact DNA methylation since this could be due to the change in expression of a partner TF required for the first TF to bind. Additionally, TF motifs enriched in DMRs could arise from TF regulation in cis, where mutations in motifs could affect TF binding leading to DMRs, which we did not investigate in this study.

The results in CpG-rich hyper-methylated DMRs consistently identified CpG-rich low complexity motifs such as EGR1 or KLF motifs in most cancer types. However, experimental validations for those TFs, investigating the impact of their loss on DNA methylation, remains challenging since they bind to CpG-rich gene promoters bound by many other TFs that might still maintain the regions unmethylated in their absence. Nevertheless, we do speculate that loss of TF binding does drive hyper-methylation in cancer.

The results in CpG-poor hypo-methylated DMRs predicted TF members of the AP-1 family (motif cluster JUN/FOS) in most cancers, those TFs regulate several important cellular functions such as proliferation, differentiation and apoptosis [37], and their role in tumorigenesis is well established [29]. In line with this, others studies have previously highlighted an enrichment of the AP-1 motif in hypo-methylated DMRs in colorectal cancer [3, 38].

We also identified other TFs that were more specific to each cancer type. Importantly, none of those predicted TFs have prominent CpG in their motifs (besides GRHL1 and CEBPB) making them good candidates to be insensitive to DNA methylation and therefore regulators of DNA methylation patterns [11]. Although many of those TFs are known to be involved in cancer, none have been shown to regulate DNA methylation patterns.

Our approach differs from previous ones as it focuses on predicting TF-binding sites that affect changes in DNA methylation independently from their association with target gene expression. It integrates the expression of TFs only to refine the list of potential candidate TF motifs. Although limited by the availability of TF motifs, we could already investigate 1048 distinct TFs out of an estimated 1600 TFs in the human genome [28]. Although we do not expect all TF-binding motif occurrences in a genome to be bound in a specific condition, we did not limit our approach to fewer experimentally derived TF-binding sites and instead performed an enrichment approach that controls for background motif occurrences. Our approach recapitulated several of the predictions from Yao et al. [13] such as the AP-1 family TFs (JUN/FOS) in several cancer types, FOXA1 and GATA3 in BRCA, NFE2L2 in LUSC, FOXA2, SOX17, and LEF1 in UCEC, and CEBPB, SPI1 and IRF7 in KIRC (Additional file 1: Figure S1 and S2). Although Rhie et al. did not integrate TF motif or binding sites, FOXA1 and GATA3 in BRCA were identified in their study [15]. The method from Fleischer et al. had also associated FOXA1 and GATA3 with changes in DNA methylation patterns in BRCA [16], which they confirmed in a recent study across 19 cancer types [39].

Based on our computational predictions, we chose to validate experimentally if binding of two candidate TFs FOXA1 and GATA3, were indeed upstream of DNA methylation changes. Previous studies also observed a local DNA hypo-methylation at FOXA1 binding sites [40, 41], which was recently confirmed by Lemma et al. [39]. Both TFs have been described as pioneer TFs as they can bind and open closed chromatin [42]. Moreover, since they do not contain CpG in their motifs, they are less likely to be repressed by DNA methylation [11], which make them good candidates to drive hypo-methylation in breast cancer.

Due to their pioneer function, they were shown to be involved in hormone-driven cancers by facilitating the access of nuclear receptors to their DNA response elements [42–45]. In breast cancer, FOXA1 and GATA3 are functionally linked with estrogen receptor alpha (ERα), and high level of expression of all three strongly correlates with the luminal subtype of breast tumors also referred as ER positive tumors (ER +) [46, 47]. Importantly FOXA1 and GATA3 binding and pioneer function are largely independent of estrogen signaling suggesting an involvement of both TFs in ER negative (ER-) tumors as well [48, 49]. We found that BRCA hypo-methylated DMRs called in ER + or ER- cancer samples compared to healthy samples were both enriched in FOXA1 and GATA3 motifs (motif *p*-value 0 for both motifs in ER + DMRs and < 10^–19^ and < 10^–9^ for FOXA1 and GATA3, respectively, in ER- DMRs).

Most FOXA1 and GATA3 binding sites in HCC1954 cancer cells were located in low methylated regions. Surprisingly, few were located in fully methylated regions but might be due to the arbitrary definition of the 200 bp window around peak summit that might not fit well the specific hypo-methylated region at some loci.

Last we tested the consequence of TF binding on DNA methylation patterns by deleting FOXA1 or GATA3 in HCC1954 cancer cells. Although most changes in DNA methylation did not occur at direct FOXA1 and GATA3 binding sites, which might result from indirect effects, we did observe a gain of DNA methylation at direct FOXA1 or GATA3 binding sites showing that FOXA1 and GATA3 do maintain hypo-methylated regions at a subset of their binding sites in cancer cells. Why only a limited number of their binding sites gained DNA methylation remains to be further explored. Interestingly, we did not observe any differences in motif sequence or occurrence between binding sites that gained DNA methylation or not. Alternatively, it might be explained by binding of other TFs to the same regulatory regions that could maintain the regions unmethylated upon FOXA1 or GATA3 removal. They could be TFs from the same families such as FOXC1, FOXJ2/3, FOXK1/2, FOXM1, FOXN2/3, FOXO1/3/4, FOXP1/4, FOXQ1 or GATA2/6 that are also expressed in HCC1954 cells [4] or other cooperating TFs.

We did not investigate here the mechanisms by which FOXA1 and GATA3 lead to demethylation and whether this occurs via a passive and/or an active demethylation through the recruitment of Ten-Eleven Translocation (TET) enzymes [50]. Interestingly, FOXA1 was shown to induce *TET1* expression through direct binding to its cis-regulatory elements, which in turn led to binding of TET1 to FOXA1 sites mediating local DNA demethylation in prostate cancer cells [51]. However, we do not observe FOXA1 binding sites around the *TET1* locus and *TET1* is not expressed in HCC1954 breast cancer cells nor in TCGA BRCA samples and other TETs have very low levels of expression.

## Conclusions

We developed a computational approach to identify TFs driving DNA methylation changes and applied it to TCGA cancer methylation data to predict TFs regulators in 13 different cancer types. This approach could be applied to a wide range of other DNA methylation datasets to infer TF regulators. We validated two TFs, FOXA1 and GATA3 in breast cancer cells, and found that their binding indeed mediates focal hypo-methylation. Altogether this demonstrates the crucial role of TFs in shaping the DNA methylation patterns of a genome and how their deregulation leads to aberrant DNA methylation changes in cancer.

## Methods

### TCGA methylation data

Raw TCGA Illumina Infinium® HumanMethylation450 BeadChip data were downloaded from the Genomic Data Commons repository (https://gdc.cancer.gov/). Loading of methylation was adapted from the ChAMP pipeline [19, 20], using the latest HM450 hg38 annotation, removing duplicated CpGs and non-mapping probes. The data were subsequently normalized using noob [21] as implemented in minfi [22, 23]. Principal component analysis (PCA) was then performed on the normalized methylation value of the first 5000 most variable positions, for each cancer. We retained all components whose explained variance is larger than 10% of that of the first component. Quadratic discriminant analysis was then trained on these components, aiming to separate the samples in two classes (cancer and healthy). Samples which were misclassified were discarded.

### Identification of DMRs in TCGA data

Differentially methylated cytosines were called using the R package limma [24] at an FDR threshold of 0.05 and cytosines located in exons were excluded (using the ENSEMBL annotation for Homo sapiens version GRCh38.87). DMRs were then called using a modified version of DMRcate [25]. The original implementation of DMRcate smoothes a *t*^2^ statistic and computes *p*-values using a *χ*^2^ distribution. This design implies that DMR detection is not sensitive to the sign of methylation change and DMRs could contain a mixture of hypo- or hyper-methylated cytosines. We therefore modified the DMRcate approach to smoothe a *t* statistic, compute *p*-values using a normal distribution and used a *p*-value threshold of 0.001 and parameter *λ* = 1000. We then selected DMRs or sub-regions of DMRs containing at least 2 consecutive CpGs that were constantly hypo- or hyper-methylated. We then selected DMRs with a minimum length of 200 bp, at least 20% methylation change, a starting methylation mean in healthy samples above 50% for DMRs hypo-methylated in cancer and below 50% for hyper-methylated ones and overlapping a corresponding cancer ATAC-seq peak [18] for hypo-methylated DMRs or not for hyper-methylated ones. We further defined DMRs as CpG-poor or -rich if they were located below or above the line defined by the equation *y* = − 1.4(x−0.38) + 0.51 defined empirically when we compared the ratio of observed versus expected CpG against the G + C content of each DMR. We defined DMRs as proximal or distal if their distance to the closest gene transcriptional start sites was below or above 2000 bp.

### TF motif enrichment

TF motif enrichment was computed and visualized using the TFmotifView approach [27]. Control regions were generated to have the same size than the DMRs, be located in the same genomic context (proximal/distal, CpG-poor/rich, promoter/intron/intergenic), in regions mappable by 50 bp reads (non-repetitive), not on chromosome Y and overlapped the same number of HM450 probes. TF motifs were extracted from a manually annotated review [28], leading to 4928 motifs grouped into 434 clusters (using TOMTOM [52] and the hclust function in R to define clusters with a threshold of 0.05) and representing 1048 distinct TFs. Using the motif probability matrices we computed for each motif its information content and mean G + C content Let f_ij_ be the frequency of letter i at position j. Then we define $local.IC_{j} = 2 + \mathop \sum \nolimits_{i}^{{}} f_{ij} log2\left( {f_{ij} } \right)$, the information content $IC = \sum\nolimits_{j} {local.IC_{j} }$, $GC.freq_{j} = f_{Gj} + f_{Cj}$ and mean. GC is the mean of $GC.freq_{j}$ weighted by its local information content. Motif G + C content was defined as rich (above 0.75), poor (below 0.25) or medium (in between). The hg38 genome was scanned for motif occurrences using mast [53] with a *p*-value threshold of 2^−IC^. Motif enrichments were then computed for each DMR category and methylation status by counting the number of DMRs and controls that contain at least one occurrence of a given motif. A pseudocount of 1 was added to all counts. The motif enrichment was defined as the percent of DMRs containing a given motif divided by the percent in control regions. A one-sided hypergeometric *p*-value was then computed, to test for enrichment significance. Motif enrichments were also computed by comparing, for each category, hypo-methylated versus hyper-methylated DMRs. In that case, the hypergeometric *p*-value was two-sided to test for depletion as well. Enriched motifs were defined using a *p*-value threshold of 0.001.

### TCGA expression data

Processed TCGA RNA-seq data were downloaded from the Genomic Data Commons repository (https://gdc.cancer.gov/). FPKMs were averaged for each gene across healthy and cancer samples, respectively. Differential expression analysis was performed using DEseq2 [54].

### Webserver for results visualization

The TCGA DMR, TF motif and expression analyses can be visualized on our webserver http://bardet.u-strasbg.fr/cancermethtf/. It was implemented in R using the shiny package (https://shiny.rstudio.com/). It was deployed using the open-source Shiny Server, was containerized using Docker (https://www.docker.com/) and uses Traefik as load-balancer (https://docs.traefik.io/). R shiny servers are optimized for Safari or Microsoft Edge web browsers.

### Cell culture

The cell lines used were obtained from the American Type Culture Collection. The normal mammary epithelial cells, immortalized with hTERT, hTERT-HME1 (ATCC CRL-4010) were cultured in Mammary Epithelial Cell Basal Medium (MEBM) supplemented accordingly to manufacturers (LONZA). HCC1954 cells (ATCC CRL-2328) were cultured in RPMI-1640 medium supplemented with 10% fetal bovine serum and 1% penicillin streptomycin. Cells were maintained in a humidified incubator equilibrated with 5% CO2 at 37 °C. All cell lines were tested negative for mycoplasma.

### Generation of knockout clones

CRISPR/Cas9 genome editing technology was used for generating knockout (KO) cell lines. To disrupt the *FOXA1* and *GATA3* genes in HCC1954 cells, guide RNAs (gRNAs) targeting the exon 1 of each gene listed in Additional file 1: Table S5 were designed by using the Benchling’s CRISPR tool available online (https://benchling.com). gRNAs were synthetized and cloned into the PX459-Puro v2.0 vector (Addgene, # 62988). HCC1954 cells were seeded into six-well plates to achieve 60% confluency before transfection. PX459-gRNA vector was transfected using FuGENE 6 in a 3:1 ratio (μL FuGENE 6: μg DNA) following manufacturer’s instructions. 24 h after transfection, transfected cells were transiently growth-selected in medium containing 2 μg/mL puromycin (Gibco) for 48 h to eliminate the un-transfected cells. Cells were individually isolated in 96 well plates. Individual clones were further expanded and knockout clones for *FOXA1* or *GATA3* expression were confirmed by immunoblotting and Sanger sequencing. One negative clone for FOXA1 or GATA3 were kept and used as controls (Ctrl) in the study.

### Establishment of overexpression cells

Full-length cDNA encoding GATA3 was amplified by PCR with primers flanked by EcoRI restriction sites. Subsequently, the purified GATA3 PCR product was ligated into a vector under the CAG promoter. The pCAG-GATA3 plasmid was transfected into the hTERT-HME1 cell line with Lipofectamine™ 2000 transfection reagent (Invitrogen) according to the manufacturer’s instruction. 48 h after transfection, cells were growth-selected in medium containing blasticidin (5 μg/mL) during several days for establishing stable cells.

### RNA isolation, cDNA synthesis and qPCR

Total RNA was extracted using the Allprep DNA/RNA mini kit (Qiagen, catalog #80,204). RNA was reverse transcribed using Maxima first strand cDNA synthesis kit (Thermo Fisher Scientific). qPCR was performed with the KAPA SYBR FAST qPCR kit (KAPA Biosystems) on a StepOnePlus PCR system (Applied Biosystems) using the standard curve method. We used fast PCR conditions as follows: 95 °C for 20 s, 40 cycles (95 °C for 20 s, 60 °C for 30 s), followed by a dissociation curve. The expression of target genes was normalized to the *RPL13A* gene. qPCR reactions were performed in triplicates with no-RT controls to rule out the presence of contaminating DNA. Primers for q-PCR are listed in Additional file 1: Table S5.

### Western blot analysis

Cells were lysed in PierceTM RIPA Lysis and Extraction buffer (ThermoFisher, #89990) supplemented with protease inhibitors (Roche). The concentration of isolated proteins was determined using PierceTM BCA protein assay kit (Thermo Fisher, #23227). Protein extracts were run on a 10% SDS polyacrylamide gel and transferred to a 0.2-μm nitrocellulose membrane. The membrane was blocked in TBS, 0.1% Tween-20 containing 5% non-fat dried milk at room temperature for 1 h and incubated with primary antibodies (dilution 1:1000) at 4 °C overnight. The membrane was washed three times with TBS-T, incubated with an appropriate horseradish peroxidase-conjugated secondary antibody for 1 h at room temperature, and washed three times. The signal was detected by chemiluminescence using the ECL detection reagent (Amersham, GE Healthcare). The following primary antibodies were used: anti-FOXA1 (GeneTex, catalog no. GTX100308 and Active motif, catalog no. 39837) and GATA3 (Assay Biotech, catalog no. B0933).

### WGBS

One hundred nanograms of genomic DNA were fragmented to 350 bp using a Covaris E220 sonicator. DNA was bisulfite converted with the EZ DNA Methylation-Gold kit (Zymo Research) and WGBS libraries were prepared using the Accel-NGS Methyl-Seq DNA Library Kit (Swift Biosciences) according to the manufacturer’s instructions with six or seven PCR cycles for the final amplification. The libraries were purified using Ampure XP beads (Beckman Coulter) and sequenced in paired-end (2 × 100 bp) on an Illumina HiSeq4000 at Integragen SA (Evry, France).

### ChIP-seq

Cells were cross-linked with 1% formaldehyde for 8 min and quenched by 125 mM glycine for 5 min at room temperature with gentle shaking. Cells were quickly rinsed in cold PBS twice then scraped in 5 mL cold PBS on ice and collected in a 15 mL conical tube. Cells were centrifuged at 4 °C at 1250×*g* for 3 min. Cell pellets were rinsed with 5 mL cold PBS, centrifuged at 4 °C at 1250×*g* for 3 min and snap-frozen in liquid nitrogen. Cell pellets were thawed on ice and resuspended in 1 mL lysis buffer 1 (50 mM HEPES–KOH pH 7.5 140 mM NaCl, 1 mM EDTA, 10% glycerol, 0.5% NP40, 0.25% Triton X-100) supplemented with protease inhibitors and incubated at 4 °C on a rocker for 10 min. Lysates were centrifuged at 1000 rpm at 4 °C for 5 min. Pellets were resuspended with 1 mL lysis buffer 2 (10 mM Tris pH 8.0 1 mM EDTA 0.5 mM EGTA 200 mM NaCl) supplemented with protease inhibitors and incubated at 4 °C on a rocker for 10 min. Lysates were centrifuged at 1000 rpm at 4 °C for 5 min. Pellets were resuspended in 1 mL shearing buffer (0.1% SDS, 1 mM EDTA, 10 mM Tris HCl pH 8.0) supplemented with protease inhibitors, then centrifuged at 1000 rpm at 4 °C for 5 min. Pellets were resuspended in 500 μL shearing buffer, transferred in a 1 mL covaris milliTUBE and sonicated with a Covaris E220 sonicator for 8 min with 5% duty, 140 peak incident power and 200 cycles per burst. The sonicated lysates were centrifuged at 16000×*g* for 15 min at 4 °C to pellet cellular debris. Sonicated chromatin in the supernatant was transferred to a new 1.5 mL LoBind Eppendorf tube. Immunoprecipitation and elution were performed using the ChIP-IT High Sensitivity kit (Active Motif #53040) according to the manufacturer’s instructions. The following antibodies were used: anti-FOXA1 (GeneTex, catalog no. GTX100308) and GATA3 (Abcam, catalog no. ab199428). Libraries, quality check and sequencing were realized by the GenomEast platform, a member of the “France Génomique” consortium (ANR-10-INBS-0009).

### Bisulfite sequencing

Hundred nanograms of genomic DNA were bisulfite converted using the EpiTect Bisulfite Kit (Qiagen) according to the manufacturer’s instructions. The target regions were amplified by PCR with the Platinum Taq DNA Polymerase (Thermo Fisher Scientific) using the following conditions: 20 cycles of 30 s at 95 °C, 30 s at 58–48 °C (with a 0.5 °C decrease per cycle), 50 s at 72 °C followed by 35 cycles of 30 s at 95 °C, 30 s at 52 °C, 50 s at 72 °C. The PCR products were cloned by TA cloning in the pCR2.1 vector (TA Cloning Kit, Invitrogen) and 15–30 clones were sequenced. Sequences were aligned with the BISMA software and filtered to remove clonal biases. The oligo sequences are provided in Additional file 1: Table S5.

### Sequencing data processing

WGBS reads were trimmed using trim_galore (version 0.6.4 options -q 20 –stringency 2 –clip_R2 10—-clip_R1 5) (http://www.bioinformatics.babraham.ac.uk/projects/trim_galore/) and mapped using bismark (version 0.22.1) [55]. Non-converted and duplicated reads were further filtered out using filter_non_conversion –percentage_cutoff 50 –minimum_count 5 and deduplicate_bismark. Methylation levels were extracted using bismark_methylation_extractor. DMRs were called using DSS [34] using CpGs with at least 5 reads coverage as input and selecting DMRs with at least 20% methylation change. For HCC1954 versus HME1 DMRs, we further selected the ones with a minimum length of 200 bp and a starting methylation level in HME1 above 50% for hypo-methylated DMRs and below 50% for hyper-methylated DMRs.

ChIP-seq reads were trimmed using trim_galore (version 0.6.4 options -q 20 –stringency 2), mapped using bowtie2 (version 2.3.0) [56] and selecting reads with mapping quality >  = 10. Peaks were called using Peakzilla [35]. In HCC1954 cancer cells, peaks were further filtered out due to localized genomic amplifications. We selected peaks with an input read density lower than its third quartile (0.2438) or that were tenfold enriched over the input sample.

### Genomic data analyses

All genomic analyses were performed using custom scripts in UNIX using bedtools [57] and awk and R for plots. Motif enrichment were performed using the JASPAR motifs FOXA1.MA0148.4 and GATA3.MA0037.3.

## Supplementary Information

**Additional file 1: Table S1.** Number of HM450 TCGA samples used. **Table S2.** WGBS sequencing data. **Table S3.** DMRs from WGBS data. **Table S4.** ChIP-seq sequencing data. **Table S5.** List of oligonucleotides used in this study. **Figure S1.** TF motif enrichment in CpG-poor DMRs. **Figure S2.** TF motif enrichment in CpG-rich DMRs. **Figure S3.** Distribution of DNA methylation in the WGBS samples. **Figure S4.** FOXA1 and GATA3 binding in HCC1954 breast cancer cells. **Figure S5.** Characterization of knockout cells.

## Data Availability

The TCGA DMR, TF motif and expression analyses can be visualized on our webserver (http://bardet.u-strasbg.fr/cancermethtf/). The datasets generated during the current study are available in the NCBI Gene Expression Omnibus repository under the accession number GSE167870.
